# Grand Duchy of Luxembourg: a case study of a national master patient index in production since five years

**DOI:** 10.1186/s12911-020-01178-y

**Published:** 2020-07-17

**Authors:** Raffaella Vaccaroli, Frédéric Markus, Samuel Danhardt, Heiko Zimmermann, Francois Wisniewski, Pascale Lucas, Hervé Barge

**Affiliations:** Agence eSanté G.I.E., Agence Nationale des informations partagées dans le domaine de la santé, 125, route d’Esch, L-1471 Luxembourg, Luxembourg

## Abstract

**Background:**

Unequivocal identification of patients is a precondition for a safe medical journey through different information systems (ISs) and software applications that are communicating and exchanging interoperable data. A master patient index (MPI) can facilitate this task. Being a repository of patient identity traits, a MPI allows an accurate surveillance of the patients’ “medical identities”. Up to 2014, the Grand Duchy of Luxembourg did not possess a MPI. Here, we describe our experience in the establishment of a national MPI for the Grand Duchy of Luxembourg.

**Methods:**

The different steps that were used to establish the MPI system are described. Firstly, through the identification of the suitable application and, secondly, through the implementation of the MPI to the eHealth national platform and its connection to the national health care system. In parallel to the first two phases, the identity management policies were defined and implemented.

**Results:**

Since 2014, when the MPI was integrated to the eHealth platform, we observed a continuous increase of identity profiles. At the latest update (31 December 2018), 2.418.336 identity profiles have been counted, including almost the totality of Luxembourgish residents (95.2%) as well as all the cross-border workers that are affiliated to the Luxembourgish social security system. An analysis of the identification domains connected to the platform highlighted a yearly increase in the usage rate of the identities by external applications (currently representing 70%). The evaluation of the quality of information contained in each identity profile showed low rejection rates (0.2%), indicating a high quality and a good level of completeness in regards to the required identity traits.

**Conclusions:**

This paper presents the current state of patient identity management in Luxembourg and discusses how this synergistically supports the functioning of the national electronic health record (EHR) known as DSP (from the French *Dossier de Soins Partagé*) and the Luxemburgish health care system. The here described national MPI has refined the identification of patients, leading to an improvement of their safety during their medical journey. Nevertheless, the application regularly undergoes updates to better meet the current requirements of the Luxembourgish health system.

## Background

During his care journey, a patient is assigned with a variety of identifiers that are generated by the various information systems (ISs) of the frequented health structures, their various departments and their interconnected applications [1–4]. Independent of this number of local identifiers, it is evident that a patient is a single individual and as such would benefit from a simplification that encompasses a characterization by a single univocal identity. As many other sectors, the healthcare sector is undergoing a rapid digital transformation that comes with an ever-growing amount of data and patient identifiers. While this digital evolution brings with it a share of advantages it also increases the risk of an erroneous patient identification [5]. Indeed, a reliable patient identification is fundamental to apply the latest advances in precision medicine. This is especially true for treatments and prevention protocols that are uniquely applicable to a targeted subset of patients. In these contexts, a switch between the identities of two distinct patients can lead to dangerous mistakes, including an entire plethora of adverse effects or, in the worst case, the death of a patient [3, 6, 7]. To lessen the risk it was decided to setup a shared master patient index (MPI) that enables cross-system patient identification [4]. Such a system is a prerequisite for electronic health records (EHRs). Nowadays, EHRs represent a major step in promoting healthcare by providing a digital version of a patient’s cross-system health data. Yet, to be able to grant the simplified care it aims to deliver, an EHR system needs to rely on a series of trustful coordination mechanisms that allow for a precise match between the correct patient and his or her medical records [3, 8]. Furthermore, such a system requires a high degree of flexibility as the digitalization of the medical sector is associated with a continuous addition of new data [5].

Taking into consideration all of the aforementioned factors, we developed a MPI solution with the aim of performing a reliable patient identification across multiple information systems. A MPI corresponds to an electronic database that holds the demographic information of patients, and that supports a univocal patient matching between healthcare services [9–11]. Such a system allows for the totality of the local identifiers of a patient to be regrouped within a unique master identity. In addition, patient data, which is collected across the various health structures, is automatically linked to the corresponding patient. Electronic MPIs have been used in healthcare since the 1980s [12], however, the interest in MPIs has strongly increased in recent years, due to the ever-growing digitalization of our society as well as of the healthcare sector.

Here we aim to present the approach we took as government agency “*eSanté*” to implement a national MPI for the Grand Duchy of Luxembourg, with the ultimate goal to standardize and facilitate identity management in the healthcare sector. Since 2014, the national MPI serves as a patient identification repository directory that facilitates the identification of patients. This identification repository is utilized by all of the services that are provided by the national eHealth platform, as well as a variety of health structures that are connected to it. Furthermore, we describe the whole integration process of the national MPI into the Luxembourgish national platform for eHealth. Finally, we illustrate the evolution of the application since its introduction in 2014.

### The eSanté agency

**Fig. 1:**
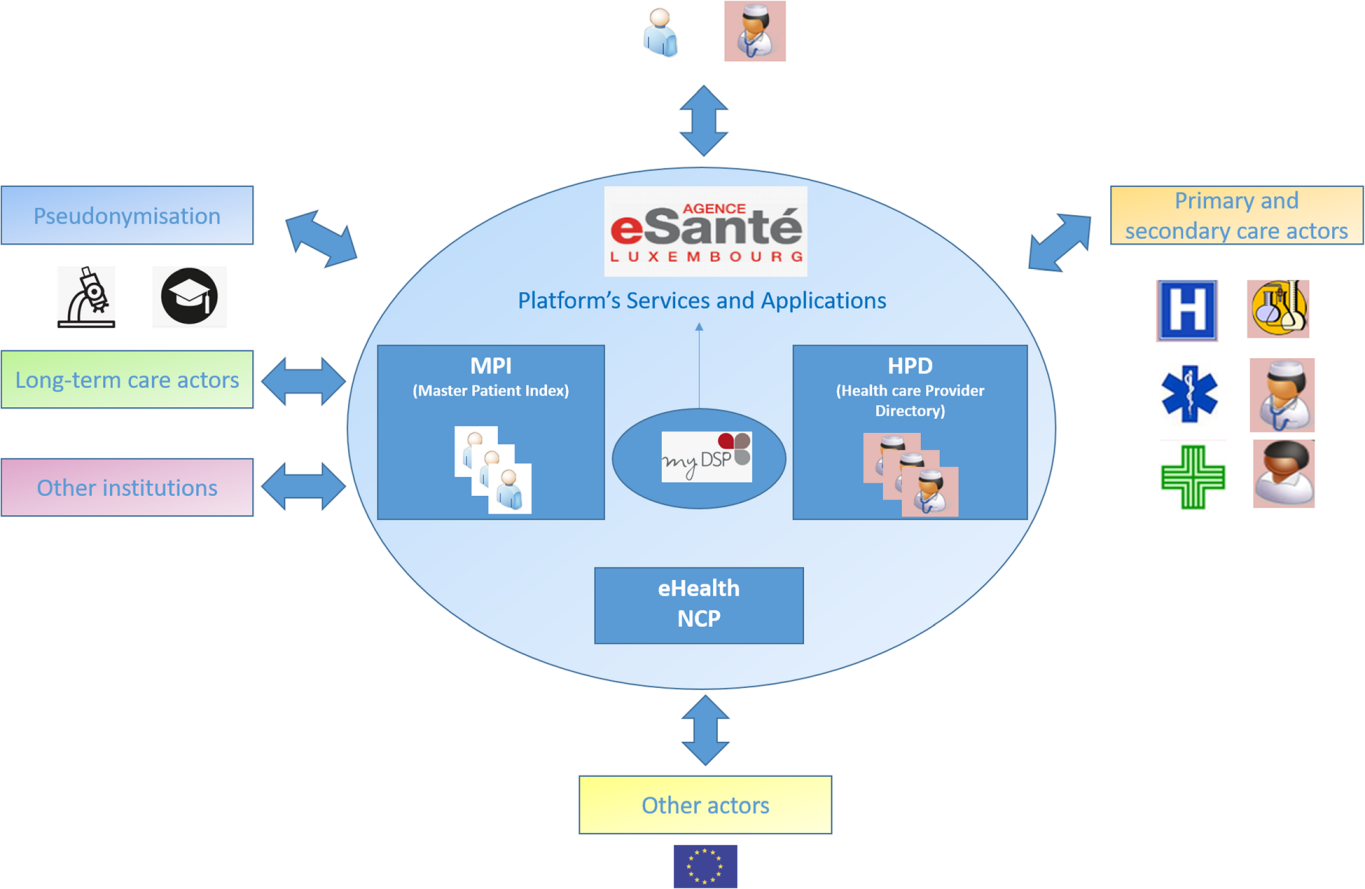
eSanté agency infrastructure and integration in the national health care system. The image illustrates some of the main services making up the eSanté platform. The core of its infrastructure corresponds to the DSP, the Luxemburgish electronic health record containing the patient’s health-related medical information. Fundamental for a reliable functioning of the DSP are the HPD and the MPI, both of which represent two key databases integrated within the platform’s architecture. The HPD contains information about all health professionals in Luxembourg as well as health institutions and structures. This directory ensures the identity of health professionals who wish to use the services of the platform to exchange medical information or to consult the DSP of a patient. The MPI directory represents the national patient identity database that enables health sector stakeholders to have a single shared view of a patient identity, regardless of the sources of identity data. Importantly, the image depicts the different healthcare stakeholders that can connect to the platform. These include primary and secondary care actors (general practitioners, hospitals, pharmacies, laboratories, […]), long term actors (care homes and nursing services) as well as others actors and institutions (European health-related institutions). Another leading service of the platform is the pseudonymization service, which is mainly aimed at universities and research institutions [4]

The establishment of a national agency for shared information in the field of health (The *e-Santé* agency [13]) was part of the of Luxemburgish “eHealth” plan [14]. Since 2014, the *eSanté* agency has been operational and managing different projects that are in accordance with the multiple missions assigned to it by law [15]. Firstly, as mentioned, a national platform for eHealth was implemented. Secondly, the platform was equipped with a healthcare provider directory (HPD) that allows for the identification of health professionals authorized to practice in Luxembourg. In parallel to the HPD, a national MPI, representing the national directory for patient demographics, was introduced. Importantly, the use of these two directories is shared by the national electronic healthcare record (the *Dossier de Soins Partagés*, DSP) as well as by all the different services associated to the platform (Fig. 1). Indeed, by virtue of the MPI and the HPD, only authorized health professionals can consult patient identity profiles. Notably, this rule applies to all the different services and health structures associated to the platform. In addition, a national strategy was defined to promote interoperability between the various health ISs and to delineate all the standards to be applied in the connections between the various health structures. Figure 1 depicts all the different directories (HPD and MPI) as well as the different internal and external applications integrated to the eSanté platform.

### The grand duchy of Luxembourg: a multicultural country

**Fig. 2:**
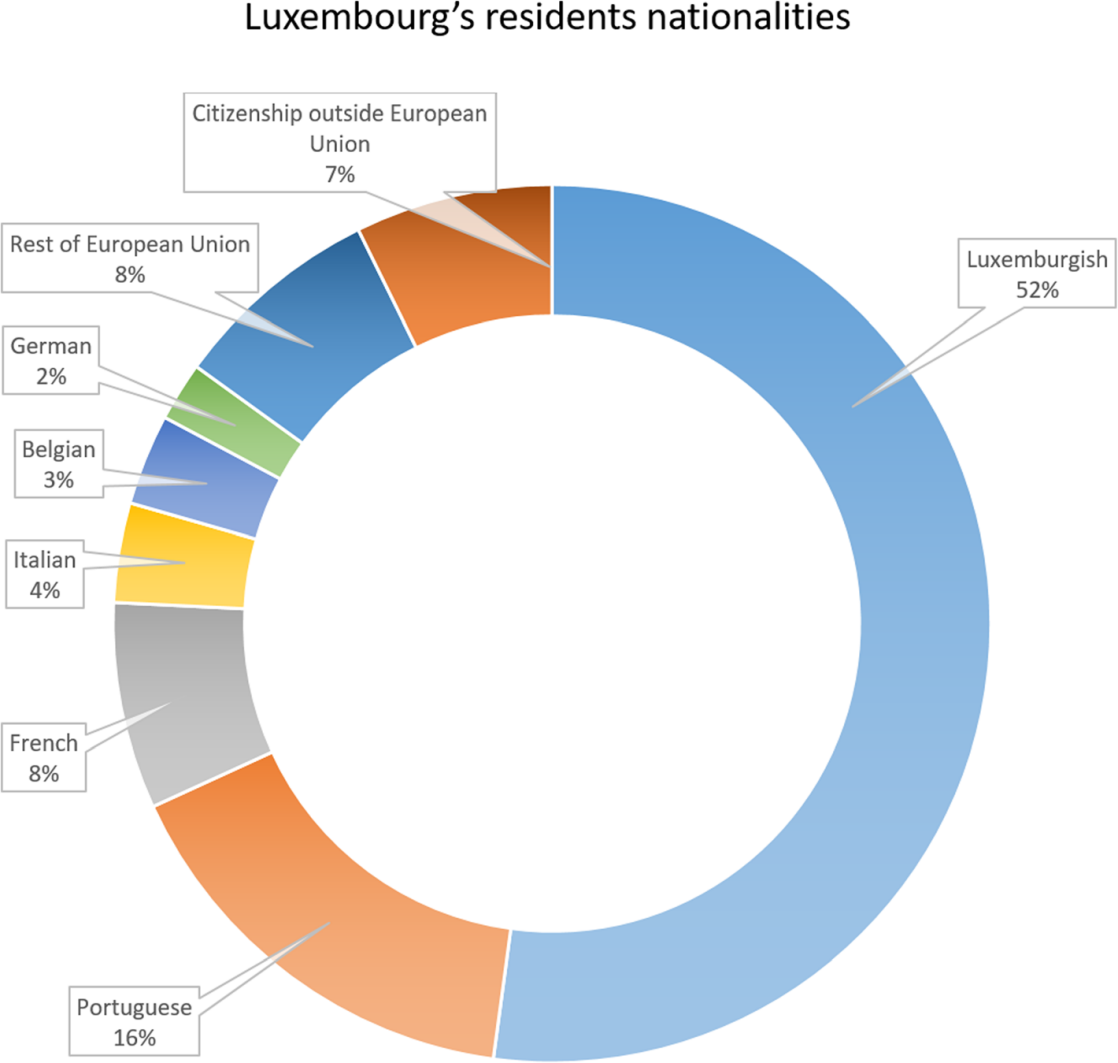
Heterogeneity of citizenship in Luxembourg. Currently, the Grand Duchy counts more than 170 different nationalities. Foreign nationals represent 48% of the Luxembourgish population. The largest community is Portuguese (33%) followed by French (16%), Italian (8%), Belgian (7%), and Germans (5%) communities. A significant part of the residents originates from other European countries (16%), or from non-European countries (15%) [16–18]

The Grand Duchy of Luxembourg with its 602,000 inhabitants represents one of Europe’s smallest countries. Nevertheless, the heterogeneity of citizenship is one of the key characteristics of Luxembourg (Fig. 2) [16]. One of Grand Duchy’s particularities is the linguistic system, which is characterized by the simultaneous use of three official languages: Luxembourgish, the national language, as well as French and German. In addition to that, other languages like Portuguese and Italian, among others, are currently being used by a great part of the population. Statistics from 2017 show that 60.2% of the residents speak two or more languages [17]. This variety had to be taken into consideration for the establishment of a successful identity management system. Another important characteristic of Luxembourg concerns the cross-border workers who, in addition to the residents, are also covered by the national social security system, bringing the total of affiliated people to 841,000 [16]. As such, all these people can benefit from the eHealth services and applications that are offered at the national level. Furthermore, Luxembourg possesses the highest demographic growth in Europe (+ 10.5% from 2014 to 2018 and a rise of about 18% is expected by 2030) [18, 19], which represents an additional factor that has to be taken into account.

### Legal bases of the MPI

The legal bases of the MPI “patient identification repository directory” are described by the article 60(3) and 60(4) of the law of the 17 December 2010 as well as by the Social Security Code [15, 20]. Three main actors are involved in the data flow regarding the identity information that is collected in the national MPI: the National Register of Natural Persons (RNPP) [21], the Joint Social Security (form the French *Centre Commun de la Sécurité Sociale* (CCSS)) [22] and the eSanté agency [13]. The RNPP generates and guarantees the uniqueness of the national identification number (Social Security Number, SSN), and keeps a record of any data updates related to the identification of a natural person in contact with a Luxembourgish administration. The CCSS, under the authority of the Ministry of Social Security, organizes the digitalization and the processing of data on behalf of various institutions (for instance Social Security, National Solidarity Fund). Demographic traits are collected by the RNPP, sent to the CCSS, which completes it with some additional affiliation dataset, and then received by the agency. In addition to this main flow, since 2016, the national identity vigilance cell (NIVC) of the eSanté agency has a direct access to the RNPP for identity verification purposes. The identity information that is gathered in the MPI corresponds to the national social security number (SSN) as well as multiple strict identity traits used to verify the patients’ identity profiles. These specific identity traits define a physical person in compliance with the amended law of the 19 June 2013 on the identification of natural persons [23, 24].

Additionally, the sharing of a patient’s identity and health information occurs in compliance with the General Data Protection Regulation (EU GDPR, enacted on May 25, 2016) [25].

## Methods

### Selection process of the national MPI solution

Our first objective was the implementation of a national MPI serving as a patient repository directory that could respond to the need of interoperability of health ISs in regard to the field of patient identification. To this end, a panorama of available MPI solutions was evaluated. It is important to note that, at the starting time of this investigation, the ideas and applications on how to ensure safety through the interoperability of local ISs for patient identification, as well as the importance of MPI solutions, were novel topics. A study published by the *groupement pour la modernisation du système d’information* (GMSIH) reviewed MPI solutions that were internationally available in 2005. The study concluded that out of the nine worldwide leading providers of ISs, only three had a MPI solution that was available [26]. In addition to this first analysis, GMSIH compared two French solutions that were applied at the regional level: STIC and Ideopass. Based on the evaluation of these documents, we opted for Ideopass, which is the precursor of the here used IdeoIdentity solution provided by Maincare Solutions [26, 27]. The choice was motivated by the capacity of the application to simultaneously act as an identity sever, enabling the management of patient identity at the central level as well as in local ISs, and to support the HL7/IHE standards recommended for identity management [28, 29].

Of note, the same solution has already been successfully integrated at the regional level by different French regions (Franche-Comté, Lorraine, Normandie, Midi-Pirénées, Provence-Alpes-Côte d’Azur, Nouvelle Aquitaine and for the PRéTDISS in Hauts de France) as well as in some overseas departments (Martinique and Guadelupe). The implementation of this application in Luxembourg represents the first example of the use of IdeoIdentity on a national level.

Since the implementation in 2014, the backbone of the application has gone through a series of modifications that were necessary to successfully accommodate the Luxembourgish legal and technical context. For instance, as recommended by the RNPP and the CCSS, the possibility to import identity data containing diacritical marks (circumflex, acute and grave accents, as well as diaresis on the five vowels a, e, i, o, u) was introduced. Another implementation that was performed concerns the introduction of an automatic daily workflow between the CCSS and the agency. This workflow is driving the import of identities or any related modifications. Furthermore, separate workflows were introduced to treat identities according to the fact that they figure or not in the CCSS identification domain (reference domain). These two workflows are described in the following paragraph.

### The national MPI

The MPI relies on a hierarchical federated identity management model [30, 31]. Such a model is composed of a dedicated centralized database collecting identity profiles that are used as references of information (central or federal identity profiles). Each of these identity profiles is assigned with a unique federated identifier (FID). The FID is only consultable by the NIVC and by the local identity vigilance cells (LIVC), and serves as a pivot identifier guiding the reconciliation of the local identifiers, which are collected by patients during their healthcare journey.

The result of this reconciliation process is a collection of local identities that are federated by a unique central identity within the reconciliation area. Furthermore, the MPI is considered to be based on a hierarchical model, since the federation and the reconciliation are performed outside of the local identification domains [30, 31].

An identification domain represents the perimeter in which the patient is represented by a single identifier. Patient identity reconciliation is consistently performed on the base of two unique identifiers (SSN and FID) in combination with a series of strict traits (first name, last name, birth date and sex) [23, 24]. The term reconciliation defines the matching, for the same person, of their two identities from two different perimeters of identification (local identification domains). We distinguish between two different workflows of reconciliation: a longitudinal one and a transversal one. The longitudinal reconciliation is defined as the reconciliation performed by identity producing domains, such as the CCSS or health facilities that are external to the eSanté platform. Furthermore, two workflows are associated to local domains performing longitudinal reconciliations. One flow is specific to the CCSS and represents a reference workflow enabled for identity injection as well as identity modification (reference workflow). Another framework applies to the other identification domains that are performing longitudinal reconciliations (secondary workflow). Indeed, an identity lacking reconciliation within the CCSS domain can be created and modified by a local identification domain performing a longitudinal reconciliation, until this same identity is reconciliated under the CCSS identification domain. For instance, when a hospital admits a patient that does not exist in the local identification domain, a request is made to the MPI to verify the existence of that patient in the national MPI. If an identity profile is present in the MPI, it is imported in the local identification domain of the hospital. Once imported, the hospital can modify the local identity profile. Of note, these modifications will be updated on the central identity profile (national MPI) only if the concerned profile does not have a CCSS reconciliation (secondary workflow). On the other hand, modifications to the central identity profile are blocked by the system in the case of an existing reconciliation in the CCSS identification domain (reference workflow). This means that, as soon as an identity profile possesses a reconciliation under the CCSS identification domain, only this domain has the right to modify the central identity. This method allows for the identification of patients whose identification traits have not been injected by the CCSS into the MPI.

On the other hand, transverse reconciliations are defined as reconciliations performed by domains that are considered as consumers of identities. Typically, these correspond to the services that are provided by the eSanté platform.

### Identities matching through a deterministic algorithm

**Fig. 3:**
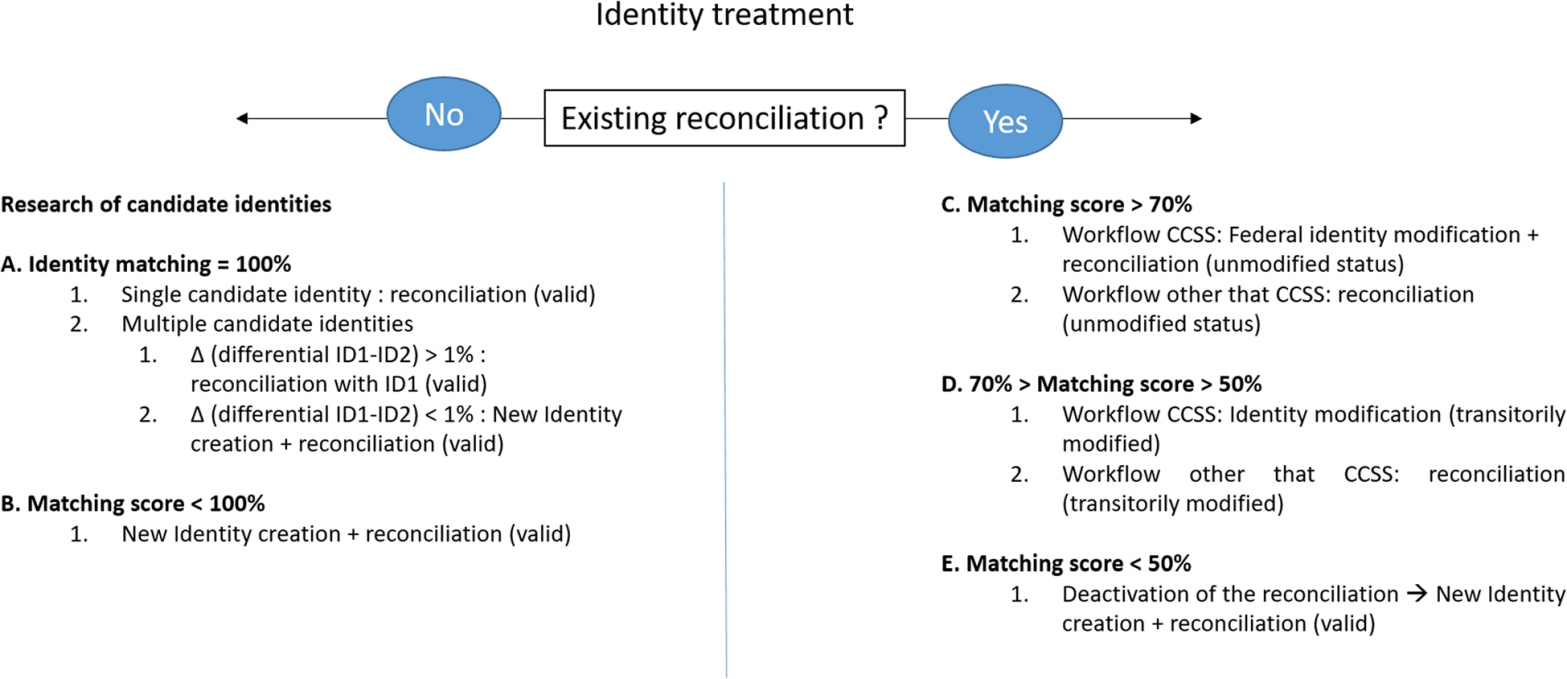
Creation and reconciliation of patient identities across identification domains: scheme synthetizing the deterministic matching algorithm functioning. The figure illustrates the functioning of the matching algorithm

A deterministic matching algorithm is used to facilitate the treatment of the injected identities and to connect the different local identifiers to the correct federal identity profiles (reference identity profiles that are contained in the MPI). This algorithm deterministically matches the traits of preexisting identity profiles (federal identity profiles on the identification server) with the information collected from the systems that are connecting to the MPI. Of note, a deterministic matching based on SSN and strict traits (Table 1) has been implemented, since these are consistently collected and represent information mandatorily belonging to an identity profile. This means that the received data elements like name, surname, birth date and sex are collected and are used to search for an exact match in the preexisting central identity profiles database. Results are represented by an overall match score expressed in percentages. In function of the different workflows, the algorithm classifies the treatment of these percentages by triggering different actions on the system. Figure 3 illustrates the identity treatments that are put into practice in relation to defined matching scores. Each time identity information reaches the identity server from a local identification domain, the algorithm starts a system search in order to understand whether, in the specific domain, there is a preexisting reconciliation for that identity. In case of a non-existing reconciliation for the analyzed identity within the specific identification domain (Fig. 3, left side), the system searches for candidate identities in the central identity server. If a search brings up a single candidate identity having a matching score equal to 100% (Fig. 3a1), a valid reconciliation is created. In the case where a system investigation leads to two or more candidate identities (ID1 and ID2), a differential (Δ, where Δ = ID2-ID1) between these two identities is calculated. If Δ > 1% (Fig. 3a2.1), a valid reconciliation with ID1 (higher score) is performed. If Δ < 1% (Fig. 3a2.2), the system creates a central identity and performs a reconciliation that is automatically considered as valid. This action creates a duplication of identity that will be highlighted by the system and that will subsequently be treated by the NIVC. In all the cases where the system research leads to a matching score of < 100% (Fig. 3b.1), another federal identity profile is created with a parallel valid reconciliation. On the other hand, for a local identity carrying a modification that was made after the previous reconciliation in a specific identification domain (Fig. 3, right side), the system maintains the reconciliation in the same status as it used to be before, with this being valid for matching scores > 70% (Fig. 3c1.2). For this last possible case, a modification of the central identity occurs only if the treatment is performed by the CCSS workflow (Fig. 3c1.1). With a matching score value between 70 and 50%, a reconciliation is labeled as transitorily modified (Fig. 3d1.2), meaning that they are treated directly by the NIVC. Similarly, the modification of the central identity is temporarily modified in the case of a treatment performed by the CCSS workflow (Fig. 3d1.1). Matching score values of < 50% are triggering a deactivation of the existing reconciliation and lead to the creation of a new central identity and a valid reconciliation (Fig. 3e).

**Table 1:** National Chart for Identity Matching

	Traits	Number of characters	Formats	Mandatory traits	Comments
**Identifiers**	National Identifier (SSN)	13	Chain of character	M	
	MPI federated identifier (FID)	10	Chain of character	M	
**Strict** **Traits**	First Name (or Given Name)	100	Chain of character	M	
	Middle Name	70	Chain of character	NM	
	Last name (or family name)	70	Chain of character	M	Multiple names are separated by a space
	Birth date	NA*	DD/MM/YYYY	M	
	Sex	1	M, F and A**	M	
**Extended** **Traits**	Birth place	50	Chain of character	NM	
	Native country	3	Chain of character	NM	Code ISO 3***
	Legal/permanent address (first line)	65	Chain of character	NM	Number (5), Street (60)
	Legal/permanent address (second line)	50	Chain of character	NM	Extension
	Correspondence address (first line)	65	Chain of character	NM	Number (5), Street (60)
	Correspondence address (second line)	50	Chain of character	NM	Extension
	Death indicator	1	Chain of character	NM	
	Date of death	NA	DD/MM/YYYY	NM	
**Complementary Traits**	Mobile phone	20	Chain of character	NM	
	E-mail	20	Chain of character	NM	

The table offers a synthetic view of an identity profile on the eSanté platform. Every physical person is associated with an internal unique identifier, the FID, as well as with the SSN. This scheme presents the complete list of demographics that were specifically collected under the strict traits, extended traits and complementary traits classifications. For each trait, the lengths of the character chain and the accepted format are illustrated. The specific format Day/Month/Year (DD/MM/YYYY) is used to specify dates. As mentioned, all the strict traits are mandatory (M), with the only exception being the middle name, which is a non-mandatory (NM) trait

*NA: Not available

**M: Male, F: Female, A: Other (from French “Autre”)

***Code ISO 3: International Organization for Standardization (ISO) ISO 3166-1 alpha-3 [48]

The validation of the matching algorithm was performed through a subset of 42 profiles, to test the identity creation, and an additional 42 profiles to verify the identity modification process.

### Requested standards and prerequisites for the interaction with the MPI of the eSanté platform

Although MPIs are mainly meant to receive patient information from various sources, the here described MPI was established with the possibility to get notifications and to provide primary systems with updates regarding patient information. To assure a bi-directional and interoperable flow of information, IHE profiles are applied [32]. These profiles correspond to a common language that integrates the needs of healthcare sites and the capabilities of healthcare IT products. Concerning the MPI service, we apply IHE-PAM, IHE-PDQ and IHE-XCPD profiles [33–35]. Furthermore, the “Patient Identity Feed” transactions of the IHE Patient Administration Management (IHE-PAM) profile are used [33], these are also defining the rules concerning the exchange with the MPI. The IHE Patient Demographics (IHE-PDQ) allows the different applications to query the CCSS and to retrieve the demographics of the patients [34]. In the context of the European program Connecting Europe Facilities (CEF), the Cross-Community Patient Discovery (IHE-XCPD) allows the identification of patients in EU member states [35]. Image 7C depicts which profile applies to which specific source and to which destination application.

Two additional IHE profiles are more generally used by the eSanté platform. These profiles are also linked to the identity server and correspond to the IHE-PIX profile [36] and the IHE-CT profile [37]. The patient identifier cross-reference manager, supporting the IHE Patient Identifier Cross Referencing profile (IHE-PIX) [36], is used to enable the reconciliation of the local patient identifiers under a unique central identifier. Finally, all the ISs communicating with our platform are requested to synchronize with official time providing servers. This is essential for traceability purposes as well as to avoid problems concerning the validity of medical data. The time synchronizations are performed by following the specification of the IHE consistent time (CT) profile [37]. Communications with the MPI use Health Level 7 (HL7) messaging in its standard version 2.5 [38] and the supported encoding characters are limited to ISO-LATIN-1.

### The functional architecture of the platform

**Fig. 4:**
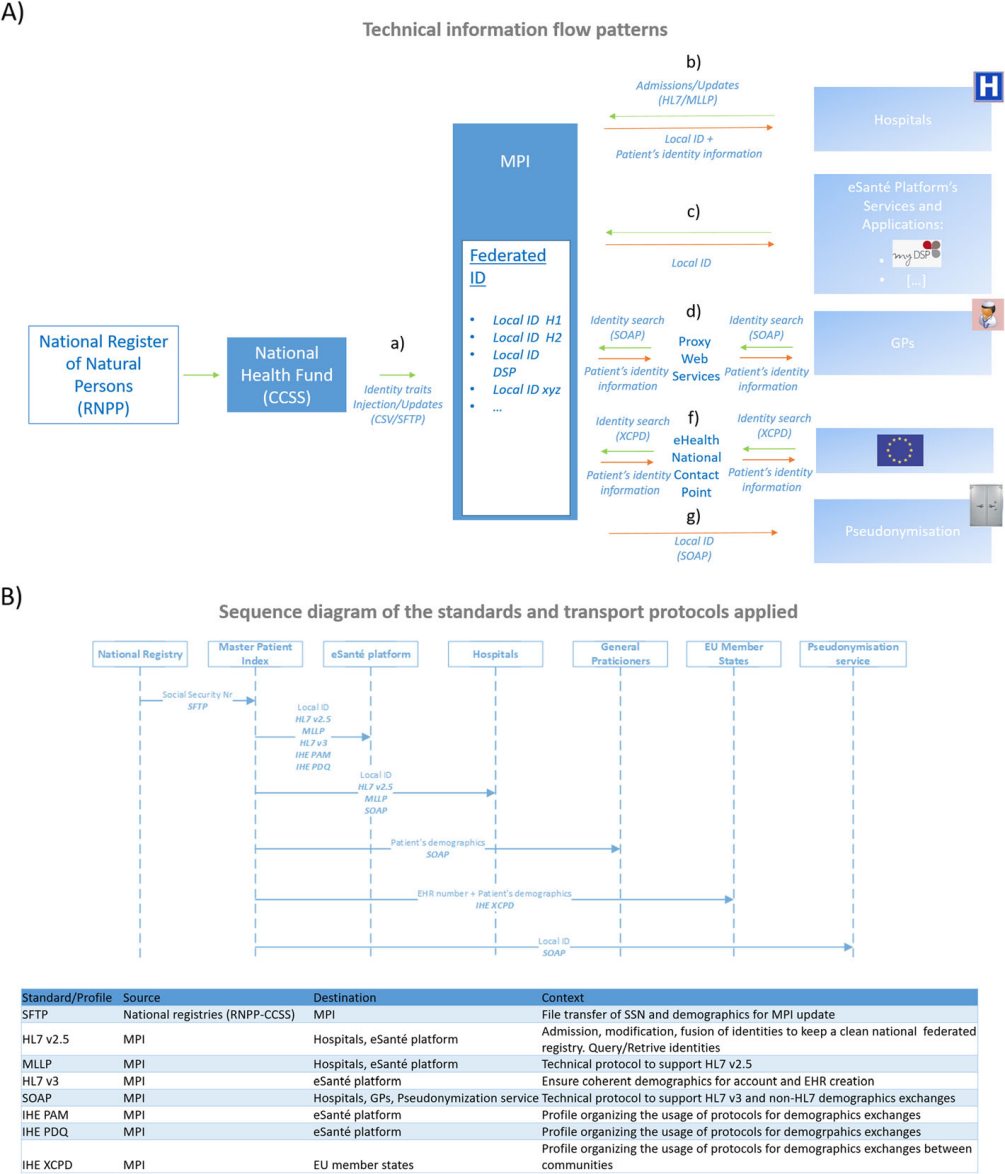
Architectural view of MPI associated to the eSanté platform. The image shows the different stakeholders of the eSanté platform and depicts how information is shared. **a**) Technical information flow patterns. The image depicts the principal stakeholders and the technical workflows applied in the process of patient identification through the eSanté platform. **b**) Sequence diagram of the standards and transport protocols that are applied. The diagram illustrates which standards and protocols are applied for patient identification by the different institutions and/or actors. A summary is given in the table underneath the diagram

In Luxembourg, the RNPP, which is established by the Government IT Centre (*Centre des Technologies de l’Information de l’Etat,* CTIE [39]), is in charge of collecting and updating all the data related to the identification of a natural person (resident or not) that is in contact with a Luxembourgish administration and affiliated to the National Heath Fund. The National Health Fund (*Caisse Nationale de Santé*, CNS), the CTIE and CCSS are in charge of creating or modifying the identities of the insured people and to provide the RNPP with the eventual updates. Figure 4a.a illustrates this initial step of the identity settlement on the identity server, which consists in the extraction of data relating to the identification of a natural person. The information is extracted from the RNPP and shared with the CCSS by using CSV files and a secure file transport protocol (SFTP). In the same way, the different departments of the healthcare structures use the minimum lower layer protocol (MLLP) as transport protocol [40] to populate the eSanté identity server, which is using the IHE-PAM profile (Fig. 4b). Healthcare providers supply the identity server with local information about the identity of patients (Fig. 4a.b). The eSanté identity integration server merges all of the services of identity research and reconciliation that are located in the different healthcare administrations, with the aim to improve the efficiency of identity vigilance politics. This bidirectional identity flow utilizes IHE web services [41] and is based on the following secured transport protocols: MLLP and the simple object access protocol (SOAP) (Fig. 4a, b-d and 7B) [42]. Furthermore, the identity integration server has the possibility to connect to a pseudonymization service that can generate pseudonyms for the identities of patients. Thus, allowing the re-use of this medical data for research purposes, which requires the patient’s consent (Fig. 4a.g). In addition, a bidirectional flow of data between the eSanté platform and the care assistance structures and home nursing services (Fig. 4a.c) has been implemented. This flow occurs with an IHE Cross-Enterprise Document Sharing (IHE-XDS) profile [43] based on SOAP transport (Fig. 4b). The same protocol applies to the various health actors that are connected to the identity server, as for example in the case of private laboratories or in the case of the applications that are used in the practices of medical physicians (Fig. 4a.g and 7A.d). Moreover, through the IHE web service-based SOAP transport, the identity server connects the identification and identity reconciliation services to the different applications and services of the platform (Fig. 4a.i and b). Furthermore, the Cross-Community Patient Discovery (IHE-XCPD) [35] profile enables the communication between different platforms that are managing the locations of a patient’s health data (Fig. 4a.f and b). Notably, this integration profile is used among European member states, involved in the deployment of Cross Border eHealth projects [44], for the identification of cross-border patients.

## Results

### A hierarchical federated identity management model

Accurate patient identification is a critical foundation to ensure data integrity and patient safety [3, 45]. To this end, one of the best-documented approach requires the implementation of identity directories, allowing unequivocal patient identification through different applications and/or health departments. To set up such a system, we decided to employ a hierarchical federated identity management model that is based on a national MPI application [30, 46]. A correct patient identification represents a key prerequisite for a reliable functioning of a national eHealth platform. For this reason, the identity management model, which we have introduced in 2014, not only applies to the services that are provided by the platform, but it also applies to each external healthcare structure connected to it. At the local level, this solution consists in the attribution of an application identifier (local identifier) and its connections with an identification domain. The identifier in question corresponds to a chain of characters and/or numbers assigned to the patient during his first contact with an identification domain. A local identification domain represents the perimeter of a single or of a group of ISs that are sharing the same local identifier to identify a patient. As nowadays patients are interacting with different local identification domains, their medical identities are generally associated with as many local identifiers as identification domains they have been using. To handle the multiple identifiers associated with a single person, in a hierarchical federation identity management model, each identity is associated with a unique federal identity profile and with an identifier that is valid at the national level, the latter of which corresponds to a 10-digit unique identifier defined as federated identifier (FID). This unique identifier is part of a national reconciliation domain that brings together the whole panorama of different local identifiers, which are collected by the various local identification domains. In general, in the hierarchical federation model that we have implemented, each time an identity is used at the local level, it is centrally reconciled underneath the federated identifier (FID) and therefore associated with the central federal identity. Anytime an identity is consulted in one of these local domains, a reconciliation with the federal identity profile (underneath the FID) is created. Each identity that reaches the MPI is analyzed by an automated matching algorithm (a detailed description of the applied matching algorithm is provided in the Method section).

**Fig. 5:**
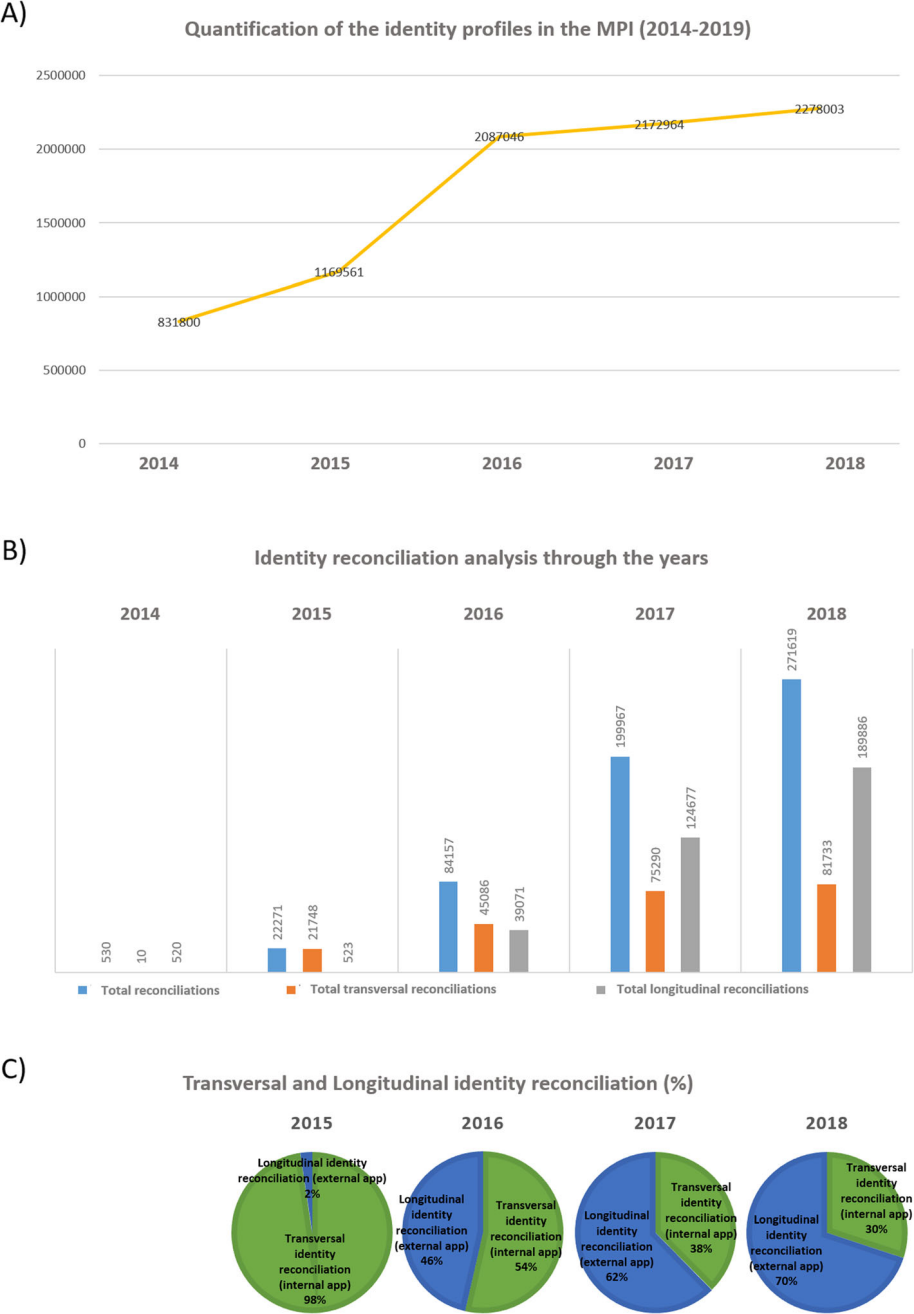
Evolution and reconciliation of identity profiles: analysis through the years (2014–2018). **a**) Evolution of identity profiles during the last 5 years. The graphical representation depicts the evolution of the total amount of identity profiles between 2014 and 2018. At the latest update (31 December 2018); the national MPI counts a total of 2.418.336 identity profiles. Data refers to the 31 December of each year. **b**) Identity reconciliation quantification. The bar chart quantifies the amounts of the different types of identity reconciliations. Blue bars illustrate the total amount of identity reconciliations. Orange bars depict the transversal identity reconciliations, meaning the identity profiles that have been retrieved by applications belonging to the platform. Gray bars illustrate the longitudinal identity consumptions by applications external to the platform. In order to perform a study about identities locally reconciliated over the years, the CCSS identification domain (reference domain) was excluded from the bar chart analysis. **c**) For each year (2015 to 2018), the pie chart depicts, in percentages, the evolution of transversal (internal application, in green) and longitudinal (external applications, in blue) identity reconciliations. Overall, an increase of the longitudinal identity reconciliations is observed

To date, 12 different identity domains are actively connected to the central MPI: seven identity domains corresponding to our platform’s services, an internal test domain, a reference domain corresponding to the CCSS identification domain and three external identity domains matching the health structures connected to the national MPI. Figure 5b shows a quantification of the reconciliations of identities that were recorded on the MPI application over the timespan of 2014 to 2018. The graphical representation depicts a very modest consumption of identities in 2014, the year when the MPI entered into production, and in 2015.These observations are linked to the fact that during the first 2 years, the identities were exclusively reconciliated by internal applications. Starting in 2016 and more prominently in 2017, an important increase in the total number of identity reconciliations is observed. Importantly, the number of longitudinal reconciliations (external application) of identities increased over the years and now represents 70% of the total identity reconciliations. This mirrors the expanding interest of external parties to connect to the platform and to benefit from a standardized patient identification process.

### Standards for patient identification: definition of an identity profile

To identify the essential elements defining an identity profile we based our work on two main references: a GMSIH publication on patient identification [1] and the national law on the identification of natural persons [23, 24]. Following the GMSIH recommendations, we defined an identity profile as the ensemble of two unique identifiers: the national social security number (SSN) as well as a central identifier, the federated identifier (FID). In addition to these two unique identifiers, an identity profile is composed of strict, extended and complementary traits, which are additional information allowing for an unambiguous identification of a person (Table 1). The MPI application rejects the identities that do not have all the requested strict traits.

Composed of 13 digits, the Luxembourgish SSN is assigned to every insured person, co-insured person, pensioner as well as people receiving a replacement income. Furthermore, children born in Luxembourg are automatically co-insured on the insurance of their parents. While the SSN identifies a person using a few digits, it also provides poor details regarding the identity: the year (four digits), the month (two digits) and the day of birth (two digits) are constituting the major part of the SSN. Additionally, to avoid duplicates, this identification number is complemented by a unique sequential range (three digits) and by two different control numbers (two digits), which are generated by two separate algorithms [23]. The SSN is issued on a card that is required for all medical services in Luxembourg. This card is automatically created when the insured person is affiliated with the CCSS. The RNPP is in charge of generating the SSN and ensuring its uniqueness, as well as of recording and updating any relevant information regarding the identification of a physical person. The SSN is defined as personal data and protected as such. It is however accessible to health professionals as well as a series of establishments, services and organizations active in the health care sector, where it is used as an identification and documentation tool. In addition to the health care sector, this identifier is widely used for identification purposes by various governmental administrations, banks as well as by private insurances. Used alone, the SSN is not suitable for primary identification as it has no photograph or any other physical description of the person. Furthermore, due to the fact that the SSN was not explicitly developed as a specific identifier for the medical sector, and following the general European and international approaches [45–47], we decided to utilize a second unique identifier (FID), which has been specifically established for the identification of patients in healthcare related matters. Additionally, the existence of a second unique identifier is fundamental to allow the identification of the minority of people that do not have a SSN. The FID is a 10-digit number randomly assigned by the MPI application once an identity profile is added. By virtue of this unique identifier, local identifiers linked to a person in distinct local identification domains can be reconciliated under the same identity profile. Of note, the FID is exclusively utilized by our MPI application and not shared with other healthcare actors or structures. Furthermore, only the NIVC and the LIVC have the rights to access this information.

As mentioned previously, together with the SSN and the FID, an identity profile is defined by a set of traits: the strict traits, the extended traits and the complementary traits (Table 1). Usually, strict traits are sufficient to identify a patient. However, if necessary, extended and complementary traits can be used to verify a given identity. It has to be noted that similarly to the SSN, the aforementioned traits correspond to confidential information that is only accessible to authorized professionals. We selected the traits using the national law on the identification of natural persons as a reference [23]. There are differences between the set of traits proposed by the MPI application and the one we implemented with the specific classification. The reason why some of the traits proposed by the application were not retained is associated to the fact that they were initially developed for the French legislation and territory. One example consists in the non-applicability to Luxembourg of codes such as the ones of the French National Institute for Statistics and Economic Studies (INSEE). Another example concerns married women, which are having the right to use their husband’s family name in everyday life. In Luxembourg, the name of use acquired with marriage is generally referred to by the words “wife of … ”. The name of use does not appear in official documents and is not entered in the civil status registers. However, the lack of standardization together with the use of alternative last names for identification can create identity issues. To remedy this problem, we opted for a unique trait labelled as “last name” where multiple names can be added.

### History

As mentioned, since 2016, our MPI application also utilizes the 13-digit SSN to perform the tasks associated with the identity verification management. In addition to the SSN, the identity profiles are completed by the different traits that are injected from the RNPP to the MPI (Table 1 depicts the detailed list). Usually, strict traits (first name, last name, sex and birth date) represent the minimum information that is required to uniquely identify a patient. However, if necessary, extended and complementary traits can be exploited for the purposes of identity verification. As such, complementary traits can be used to provide medical, administrative or additional information. In case of a doubt, these are information that can be consulted by the NIVC or by authorized professionals.

**Fig. 6:**
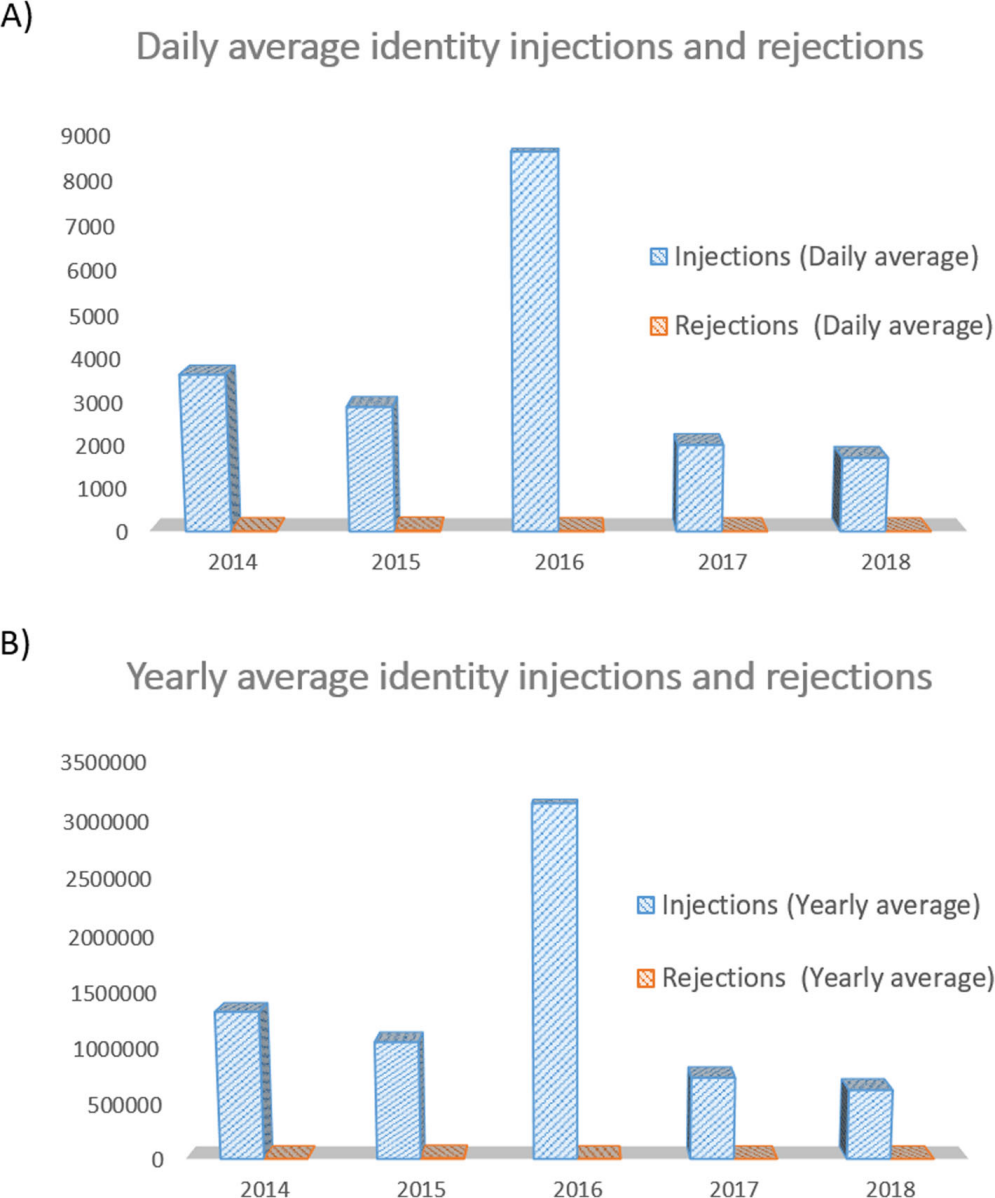
Average number of injections and of rejections of identity profiles through the years (2014–2018). **a**) The graphical representation illustrates the daily average number of identity profiles that have been injected (in blue) or rejected (in orange) daily between the years 2014 and 2018. **b**) The graphical representation depicts the annual average of identity profiles that have been injected between the years 2014 and 2018. The average of annual rejections is shown in orange

The first injection of patient identity profiles into the national MPI was carried out in 2014 by using the CCSS data as reference source. From the CCSS database, 776.378 identities were transferred during this initial injection (Fig. 5a, 2014). Notably, in Luxembourg, 95.2% of the population is affiliated to the CNS [16]. This means that the majority of the identity profiles corresponding to residents as well as cross-border workers, which are also affiliated to the national health fund, are represented in the patient identification repository directory. Furthermore, for this first import the selection was limited to identity profiles having a 100% completeness regarding the strict traits, and for which the insured and co-insured status was reported as valid. The following injections were consisting of daily updates and “manual” imports of identity profiles, as well as “manual” controls performed to correct erroneous data imports (for example caused by format problems). Since 2016, this workflow has been fully automatized. As such, the information contained in the RNPP is initially transferred to the CCSS. In a second step, the CCSS automatically injects the information into the patient identification repository directory (Fig. 4a and b). Importantly, the flow of information occurring between the RNPP and the MPI realizes not only the import of identity data but also any eventual update concerning the same data. The CCSS/CTIE database keeps daily track of demographic updates related to the residents, thus relevant new information can be instantly incorporated into the MPI. The same updates occur periodically for cross-border workers. In this interconnection, daily identity updates and/or modifications are shared using a SSH File Transfer Protocol (SFTP) and through a so-called “Delta file”. This process allows the daily review of around 2000 identity profiles, firstly in a test environment and ultimately in a production environment (Fig. 6a). This double step ensures a quality control of the imported data. As an additional measure to ensure the trustworthiness of the information contained in the MPI, our NIVC has access to the National Register of Natural Persons, which regularly updates the CCSS database on which the MPI is based. This access is rarely used and is limited to the NIVC. The access to the RNPP by NIVC is only foreseen to facilitate the treatment of identity issues when a doubt remains, or for verification purposes of specific traits that can change over time like the “last name” of a woman.

During the course of the 5 years of development and functioning, the national MPI has counted the passage of more than 6 million identity profiles. These were injected either for creation purposes or for update purposes (annual average of 1.3 million, Fig. 6b). Most of this identity profiles have been reconciliated to a preexisting central identity. Alternatively, certain identity profiles have been injected to update the information of existing federal identity profiles. These identity profiles are not counted as unique patient identities but only as injections. The number of unique patient identities contained in the MPI corresponds to 2.278.003 (as of the 31st December 2018, Fig. 5a).

In this process, the yearly rejection rate represents only 0.2%. This minor percentage reflects the set of identity profiles lacking specific strict traits, for instance the trait “Name”, which is considered as essential for an identity profile to be accepted into the MPI application. Indeed, this strict trait does not exist in various countries, or is not required for purposes of identification. We are presently cooperating with the software provider on a solution that will allow us to accept these identities.

### Vigilance on identities

**Fig. 7:**
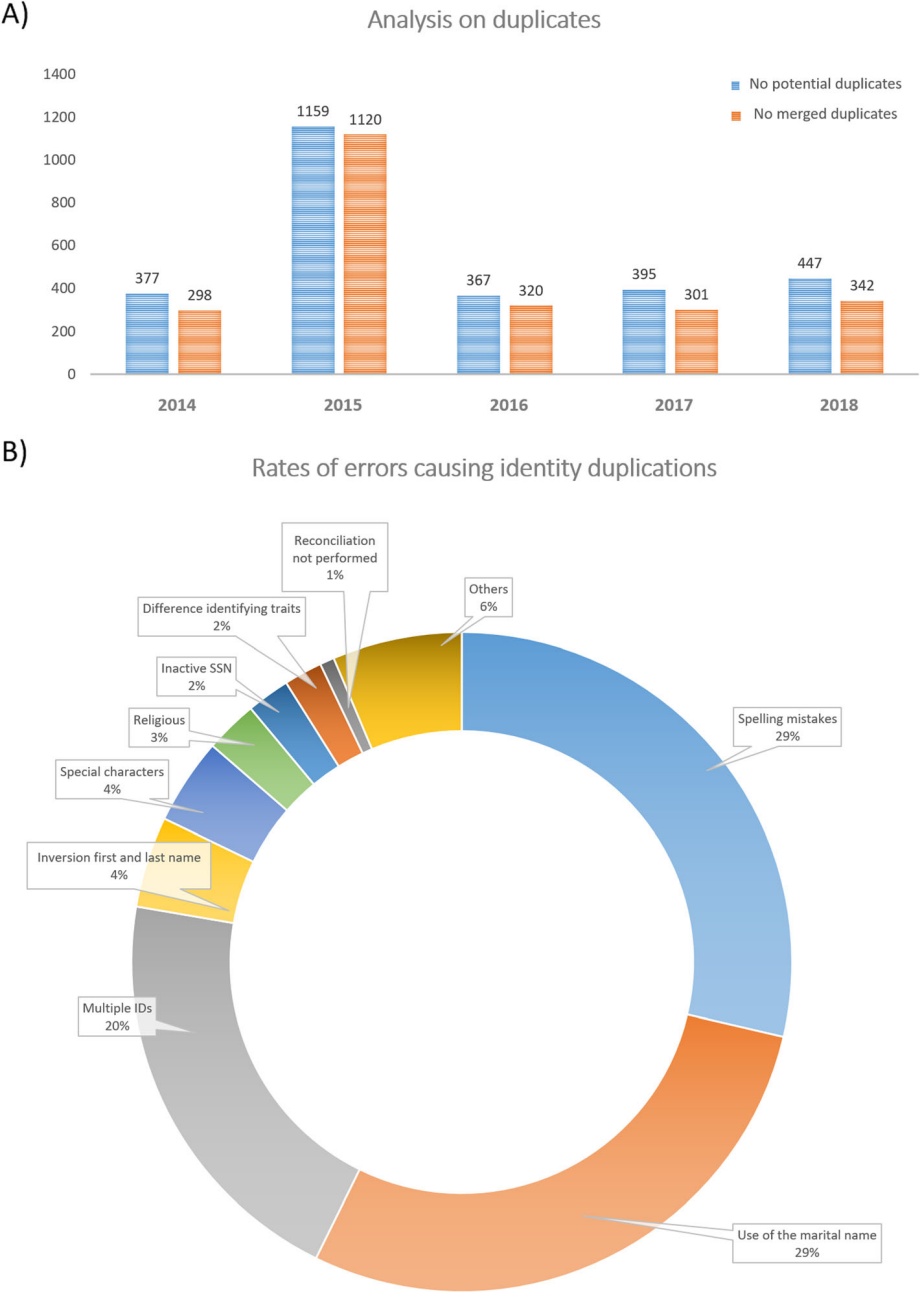
Analysis on identity vigilance. **a**) Analysis on the number of duplicates. The graphical representation illustrates the number of potential duplicated identities (in blue) and the number of merged identities (in orange). The depicted values represent the sum of cases that occurred within the indicated year. **b**) Rate of errors leading to identity inconsistencies. The pie chart reports the most common errors leading to identity duplicates, where the percentage corresponds to the rate of occurrence. The term “others” refers to the ensemble of various errors that were only reported one or two times

With the connection to the patient identification repository directory, healthcare structures and services aim to assure the safety of a patient through the reconciliation, into a unique identity, of the entire array of information that is collected by different identification domains. As previously mentioned, the core problem resides in identities that are falsely categorized and thus prevent the critical information from reaching medical records, and as a consequence, health providers during a care visit. The functioning of our patient identification repository directory is guaranteed by the daily work of our NIVC. To date, the NIVC is composed of two operators. In particular, the NIVC performs a daily control on the identity data coming from CCSS, with a particular focus on investigating potential errors that were identified by the application (for example format of the source not compatible with the used standards). Furthermore, the NIVC is responsible of the validation of a pre-treated process revealed by the deterministic matching algorithm. Importantly, only the NIVC possesses the rights to investigate and to solve identity issues and, as such, is the one in charge of resolving identity inconstancies. Of note, in a hierarchical identity federation model, one of the most common example of identity inconsistency concerns duplicates. These correspond to situations where two or more FIDs, and therefore central identity profiles, relate to the same person. A major risk associated with identity duplicates is information spreading between two identity profiles. As this can lead to alternative associations of information to either one of the affected identity profiles, a possible consequence could be the loss or confusion of medical information, which in turn could lead to inadequate medical decisions. In order to reduce these inconstancies in the identity profiles, we have complemented the system with a deterministic matching algorithm. As mentioned in the method section (Identities matching through a deterministic algorithm), this algorithm facilitates the identity management by creating non-existing identities, and by spotting false or ambiguous patient matching through the use of strict traits (first name, last name, birth date, sex) and the use of the SSN. Once the algorithm spots a potential duplicate, the NIVC is notified and is in charge of the final analysis. Duplicates are initially qualified as a “potential duplicate”, due to the indications that the concerned identities could correspond to the same person. This step is followed by the analysis by the NIVC and once the NIVC has treated the issue, and there is no more doubt about the subject in question, the duplicates are labelled as “merged duplicate”. In this case, the processing of duplicates consists in merging identities, one acquiring the status of master identity and the other of slave identity, the latter of which will then be deactivated. Any further research regarding the slave identity will point to the master identity [7, 49]. Figure 7 depicts the rate of potential duplicates and merged duplicates that occurred during the 5 year development period of the national MPI.

Collisions are another form of identity issues that can occur. In this case, two (or more) people are assigned the same central identity profile. Such mergers of identities are mainly detected in care structures while investigating anomalies in the medical data. Following their detection, healthcare professionals report these collisions to the NIVC. To date, the number of collisions detected by the MPI is equal to zero. In general, when in doubt, good practice in terms of identity vigilance requires the creation of a new identity (and therefore a possible duplicate) rather than to risk a collision. This approach has been implemented in the configuration of the identity matching algorithm, in such a way that a matching is created only if the correspondence rate for strict features is equal to 100%.

Importantly, the NIVC personnel is trained directly by the MPI software company. Furthermore, two different working groups are in place. The first one brings together all the users of the IdeoIdentity system. The aim of this group is to share the experience about the service with the users and with the software provider. Furthermore, the software provider utilizes this working group to announce and to train users in the new versions of the application. The second working group is composed of the NIVC and the LIVCs, which are the representatives in charge of handling identity vigilance issues locally. This second group is tasked with the mission to discuss and elaborate the local and national exigences concerning identity management, eventually resulting in the proposition of improvements that can be applied to the application.

To date, the patients’ identity directory contains 2.278.003 identity profiles (Fig. 5, latest update on the 31/12/2018). One of the main healthcare structures in Luxembourg has retrieved 180.266 identity profiles since their first connection to the MPI application, which represents 8% of the total recorded identities within the MPI. For this specific structure, 400 identity profiles are treated per day, with 426 identity anomalies being reported for the entirety of 2018, which corresponds to 1.2 anomalies per day as well as 0.3% of the average of total identities retrieved daily. Figure 7b reports the rates of the most common sources of error leading to duplication of identities. In more detail, 29% of the described anomalies are due to the alternative use of marital and family name, other 29% are associated with spelling mistakes, and 20% of the duplicated identities have been reported to be associated with the existence of different local identifiers.

### Foreseen evolutions

Between the initial establishment and the current management of the MPI, we have requested several modifications to the application. These modifications are necessary to further adapt the application to the Luxembourgish legal and technical context. As such, we are currently working on the possibility to add information regarding the affiliation and filiation to each identity profile. Another important step we took to improve the MPI was the acquisition of a module allowing us to delegate the management of duplicates to the LIVCs. Importantly, due to this specific module the health structures, which are in direct contact with patients, are allowed to perform a decentralized pre-treatment of identity anomalies and to upload this information to the national MPI.

### Secondary use of patient identity management for translational research

Healthcare professionals collect various types of data during patient care that could help clinical and academic research institutions [50–52]. In this context, emerging legislations are pushing the need to protect the privacy of the patient in regards to their medical data. For example, the European General Data Protection Regulation (GDPR) [25] mirrors, through the article 25 “Data protection by design and by default” [53] and the article 32 “Security of processing” [54], the European will to preserve confidentiality while handling the health data of patients. Different strategies allowing for an ethic use of the patient’s confidential medical data are currently materializing, among which is the approach of pseudonymization. As the name suggest, the real identity of the patient is replaced with a pseudonym. Within the eSanté platform, pseudonymization is performed through the use of a specific service, the Service Pseudonymization Santé (SPS). With the pseudonymization service we aim to facilitate the legislative and administrative procedures of clinical and academic research. Of note, to perform pseudonymization compliant with the current legislative requirements, another update was introduced to the national MPI infrastructure, by implementing a specific intermediary MPI that is dedicated to this application (SPS-MPI). This specific MPI assures an intermediate layer of identity management that applies exclusively to the identities that are related to services or studies utilizing the SPS. A further publication will describe the pseudonymization techniques available on our platform and illustrate the link between the SPS-MPI and the national MPI.

## Discussion

Before 2014, patient identification in Luxembourg was based on the use of the SSN in association with additional identification traits. Notably, the traits applied for identity verification were variating depending on the local context as no standardization policy was in place. In Luxembourg, the use of the SSN is similar to the personal identity number applied in Sweden [55]. As national identifier, the SSN was never designed for the specific use in the care sector, but for more general identification purposes. In contrast, international guidelines for patient identification strongly encourage the use of multiple factors in the involved identification systems [1, 3, 45, 56]. For this reason, in 2014, we not only implemented a MPI system based on a hierarchical federation model, but also introduced a unique identifier (FID) that is specifically pinpointing and linking patients in the health sector. Our federated model possesses the capacity to reconciliate the multiplicity of unrelated local identifiers underneath these FIDs. Thanks to the FID and the use of additional traits (strict traits: first name, last name, date of birth, address, sex), the identity server coordinates the flow of a patient’s medical records, all of which are coming from multiple medical institutions. As previously mentioned, this task is helped by the use of identity information that is extracted by the RNPP, and therefore represents identity information belonging to official governmental sources (RNPP and CCSS). Furthermore, the system employs a deterministic matching algorithm [57]. As explained in detail in the methods section, this algorithm performs a deterministic match that is based on comparing the set of traits that are identified as strict and the SSN. In line with international recommendations [1, 3, 45, 56], strict traits as well as SSN are used for the calculation of matching scores. These are used as they represent the traits that are consistently present within an identity profile. In the case of absence of any of these traits, the concerned identities are rejected by the system. When analyzing the most common errors that lead to identification issues (Fig. 7b), we observed that they are mainly resulting from the alternative use of marital names and family names as last official names, that they are associated with typographical mistakes or that they are due to the existence of different local identifiers. The existence of multiple identifiers can be easily traced back to the alternate use of identification traits like the last name or to typographical errors, and thus represents a straightforward problem. On the other hand, the habit of women to alternatively use their marital or their family name can vary between Luxembourg and its neighboring countries. The aforementioned typographical errors can be explained by the multiple spelling habits present in Luxembourg, which are due to its cultural heterogeneity. The results we show in this context are in line with the data documented in literature (Fig. 7b) [2, 6, 11]. For instance, the analysis on identity duplication made by Just et al. revealed that typographical errors in demographic traits and SSN were one of the most frequent causes leading to identity issues. In addition, the same study takes into consideration the constant change of demographics as a factor that increases the difficulty of patient identification, especially in systems relying on deterministic matching [11].

Overall, a complete and accurate patient identification not only enhances the coordination between care providers, but also translates to an increase in health security. Within this frame of reference, the use of automated patient identification systems for patient identification is a practical mean to reduce the risk of human error [2] . Being aware that human errors cannot be eliminated, a more intense effort should be invested in the statistical analysis of mistakes leading to identity issues. The resulting understanding would prove useful to further optimize the training of personal associated with the generation of health data. Moreover, this information should be taken into consideration when optimizing the algorithms used to handle patient identification. To date, the system we are using does not employ probabilistic matching. Nevertheless, a possible future objective could certainly be to focus on the development and implementation of more sophisticated tools to be associated with the deterministic matching in use.

## Conclusions

As previously described, our MPI management activities are based on the IdeoIdentity application. It is fundamental to underline that, since the MPI inception in 2014, we have continuously designed and implemented updated versions of the application. Thanks to these regular improvements, which were programmed by the software provider, we were able to parameter an application reflecting the national context and requirements. Currently, the database contains data on all of the Luxembourgish residents and cross-border workers that are affiliated to the Luxemburgish social security system. In addition, the majority of the actors in the healthcare sector are regularly using the platform, leading us to conclude that the system has been successfully implemented. Concerning the future evolution, we consider our MPI as a tool that can be flexibly adapted to upcoming necessities. For this reason, further implementations are planned as to assure the best possible compatibility in regards to national as well as international best standards in patient identification.

As previously described, the practical implementation of such an approach was met with a series of challenges. As the Grand Duchy of Luxembourg is a multicultural country, the associated heterogeneity of the population means different spelling habits as well as differences in phonetic transcription due to the different linguistic contexts. For this reason, the risk of generating identity anomalies is increased. Indeed, we observed that general typing mistakes (29%) together with alternate uses of marital name and / or maiden name for females (29%) represent the primary causes for reported identity anomalies (Fig. 7b). Furthermore, patient indexing is challenged by high recurrences of homonyms and hyphenated names. All these recurring errors either have been taken into account or are currently being investigated in order to deliver the necessary improvements to the MPI application as well as to our NIVC work. Apart from the technological advances that can be implemented, it is clear that flexible alignments of ISs as well as local acceptance of the results are essential to assure patient security through a proven management of identities.

## Data Availability

The datasets used and/or analyzed during the current study are available from the corresponding author upon reasonable request.

## Abbreviations

CCSS: Centre Commun de la Sécurité Sociale / Common Social Security Center
CISS: Centre Informatique de la Sécurité Sociale / Social Security IT
CNS: Caisse Nationale de Santé / National Health Fund Center
CT: Constant time
CTIE: Centre des Technologies de l’Etat / State’s Center for IT
DSL: Digital Health Infrastructure
DSP: Dossier de Soins Partagé
EHR: Electronic Health Record
EIG: Economic Interest Group
FID: Federated Identifier
GDPR: General Data Protection Regulation
GMSIH: Groupement pour la Modernisation du Système d’Information Hospitalier/Grouping for the Modernization of the Hospital Information System
HL7: Health Level 7
HPD: Healthcare Provider Directory
IHE: Integrating the Healthcare Enterprise
INSEE: French National Institute for Statistics and Economic Studies
ID: Identifier
IS: Information System
IT: Information Technology
LIVC: Local Identity Vigilance Cell
MLLP: Minimum Lower Layer Protocol
MPI: Master Patient Index
NIVC: National Identity Vigilance Cell
PAM: Patient Administration Management
PDQ: Patient Demographics Query
PIX: Patient Identifier Cross-Referencing
PRéTDISS: Regional Project of Digital Transformation of the Health System
RNPP: Registre National des Personnes Physiques / National Register of Natural Persons
SFTP: SSH File Transfer Protocol
SOAP: Simple Object Access Protocol
SPS: Service de Pseudonymisation en Santé / Health Pseudonymization Service
SSN: Social Security Number
WS: Web Services
XCPD: Cross-Community Patient Discovery
